# Prevention of DNA Rereplication Through a Meiotic Recombination Checkpoint Response

**DOI:** 10.1534/g3.116.033910

**Published:** 2016-09-27

**Authors:** Nicole A. Najor, Layne Weatherford, George S. Brush

**Affiliations:** * Department of Pharmacology, Wayne State University School of Medicine, Detroit, Michigan 48201; † Department of Oncology, Wayne State University School of Medicine, Detroit, Michigan 48201; ‡ Molecular Therapeutics Program, Barbara Ann Karmanos Cancer Institute, Detroit, Michigan 48201

**Keywords:** Mec1, Rad53, recombination, DNA replication initiation, protein phosphorylation

## Abstract

In the budding yeast *Saccharomyces cerevisiae*, unnatural stabilization of the cyclin-dependent kinase inhibitor Sic1 during meiosis can trigger extra rounds of DNA replication. When programmed DNA double-strand breaks (DSBs) are generated but not repaired due to absence of *DMC1*, a pathway involving the checkpoint gene *RAD17* prevents this DNA rereplication. Further genetic analysis has now revealed that prevention of DNA rereplication also requires *MEC1*, which encodes a protein kinase that serves as a central checkpoint regulator in several pathways including the meiotic recombination checkpoint response. Downstream of *MEC1*, *MEK1* is required through its function to inhibit repair between sister chromatids. By contrast, meiotic recombination checkpoint effectors that regulate gene expression and cyclin-dependent kinase activity are not necessary. Phosphorylation of histone H2A, which is catalyzed by Mec1 and the related Tel1 protein kinase in response to DSBs, and can help coordinate activation of the Rad53 checkpoint protein kinase in the mitotic cell cycle, is required for the full checkpoint response. Phosphorylation sites that are targeted by Rad53 in a mitotic S phase checkpoint response are also involved, based on the behavior of cells containing mutations in the *DBF4* and *SLD3* DNA replication genes. However, *RAD53* does not appear to be required, nor does *RAD9*, which encodes a mediator of Rad53, consistent with their lack of function in the recombination checkpoint pathway that prevents meiotic progression. While this response is similar to a checkpoint mechanism that inhibits initiation of DNA replication in the mitotic cell cycle, the evidence points to a new variation on DNA replication control.

DNA replication during meiosis generates the necessary chromosomal content for the subsequent formation of haploid gametes through two consecutive rounds of chromosome segregation. As during the mitotic cell cycle, meiotic DNA replication is tightly regulated so that initiation occurs at precisely the correct time, and only once during the process (Strich 2004); in the absence of appropriate controls, errors such as DNA rereplication can occur that are typically harmful to the cell. Cyclin-dependent kinase (CDK) complexes are central regulators of eukaryotic DNA replication initiation, both in the mitotic cell cycle (Siddiqui *et al.* 2013) and in meiosis (Dirick *et al.* 1998; Stuart and Wittenberg 1998; Benjamin *et al.* 2003). We have shown in *Saccharomyces cerevisiae* that expression of a stabilized form of the B-type cyclin-CDK inhibitor Sic1 during meiosis can lead to extra rounds of DNA replication (Sawarynski *et al.* 2009). This observation is consistent with the well-established role of CDK, particularly Clb5-Cdk1, in preventing DNA rereplication during the mitotic cell cycle through several mechanisms that serve to inhibit reformation of the prereplicative complex (Nguyen *et al.* 2001; Ikui *et al.* 2007; Siddiqui *et al.* 2013).

As in most eukaryotic organisms, meiotic DNA replication in *S. cerevisiae* is followed by programmed recombination between homologous chromosomes during prophase of the first meiotic division. The physical interaction of homologs afforded by recombination is important for accurate chromosome segregation during this division, and allows for transfer of genetic information between the parental chromosomes. Meiotic recombination initiates from a DNA double-strand break (DSB) generated by Spo11, a topoisomerase-like enzyme with DNA transesterase activity that functions in cooperation with several other proteins (Keeney *et al.* 1997; Maleki *et al.* 2007). It is estimated that Spo11 catalyzes formation of 140–170 DSBs per meiosis in *S. cerevisiae* (Buhler *et al.* 2007; Pan *et al.* 2011), with a number of controls in place to ensure that each of the 16 chromosomes sustains at least one event (Youds and Boulton 2011). Each DSB is initially processed to generate 3′-single-stranded DNA overhangs that can invade the homologous duplex chromosome (Cao *et al.* 1990; Sun *et al.* 1991). In the absence of the meiosis-specific DNA recombinase Dmc1, strand invasion cannot proceed and extensive DNA resection results, leading to activation of a meiotic recombination checkpoint response that prevents exit from the pachytene stage of prophase I (Bishop *et al.* 1992; Xu *et al.* 1997).

As might be expected, the meiotic recombination checkpoint pathway as defined by deletion of the *DMC1* gene (*dmc1*Δ) shares many proteins with DNA damage checkpoint pathways that operate during the mitotic cell cycle (Lydall *et al.* 1996). Examples include the apical protein kinase Mec1, and its associated protein Ddc2, which are orthologs of human ATM- and Rad3-related protein kinase (ATR), and ATR-interacting protein (ATRIP), respectively, and the PCNA-like Ddc1-Mec3-Rad17 (“9-1-1”) complex, which facilitates Mec1 function, and also has a human counterpart (Weinert *et al.* 1994; Lydall *et al.* 1996; Paciotti *et al.* 2000; Hong and Roeder 2002; Zou and Elledge 2003; Navadgi-Patil and Burgers 2011; Refolio *et al.* 2011). In addition, the Dot1 methyltransferase is involved in both (San-Segundo and Roeder 2000; Giannattasio *et al.* 2005; Wysocki *et al.* 2005). By contrast, the protein kinase Rad53, an ortholog of human CHK2, and its mediator Rad9, similar in some respects to human mediators such as BRCA1 and 53BP1, function downstream of Mec1 in various cell cycle DNA damage checkpoints (Allen *et al.* 1994; Weinert *et al.* 1994; Sun *et al.* 1996; Gilbert *et al.* 2001; Stracker *et al.* 2009), but are not involved in the meiotic recombination checkpoint (Bishop *et al.* 1992; Lydall *et al.* 1996; Bailis and Roeder 2000). While Rad53 and Rad9 have been implicated in certain meiotic checkpoints, including the response to hydroxyurea (HU) (S-phase) (Blitzblau and Hochwagen 2013), and to unprogrammed DNA damage (Weber and Byers 1992; Cartagena-Lirola *et al.* 2008), their absence in the recombination checkpoint can be explained by the existence of meiosis-specific proteins that operate specifically in the context of recombination intermediate structures (Hollingsworth and Ponte 1997; Xu *et al.* 1997; Bailis and Roeder 2000). These include Hop1, Red1, and Mek1, each of which is a component of the sister chromatid-derived axial elements that form during meiosis, and are critical for proper meiotic recombination. Hop1 and Red1 are structural in nature (Hollingsworth *et al.* 1990; Smith and Roeder 1997), whereas Mek1 is a protein kinase with sequence similarity to Rad53 (Rockmill and Roeder 1991; Leem and Ogawa 1992; Bailis and Roeder 2000). All three proteins help to enforce the proper bias of interhomolog recombination during unperturbed meiosis, thereby promoting faithful chromosome segregation (Hollingsworth and Byers 1989; Rockmill and Roeder 1991; Schwacha and Kleckner 1997; Thompson and Stahl 1999; Kim *et al.* 2010; Wu *et al.* 2010). In the context of the meiotic recombination checkpoint activated by deletion of *DMC1*, Red1 associates with the 9-1-1 complex to help activate Mec1 (Eichinger and Jentsch 2010), leading to Mec1-catalyzed Hop1 phosphorylation required for Mek1 activation (Carballo *et al.* 2008). Mek1 activity in turn prevents excessive recombination between sister chromatids, and thereby maintains the checkpoint signal (Wan *et al.* 2004; Niu *et al.* 2005). Studies using mutants with defects in intersister DSB repair, or those in which the DSBs that are generated cannot be efficiently repaired, have shown that Mek1 also serves to prevent meiotic progression (Xu *et al.* 1997; Cartagena-Lirola *et al.* 2008; Wu *et al.* 2010).

Ultimately, checkpoint-mediated prevention of pachytene exit and progression through the meiotic divisions is implemented in part through regulation of the *NDT80* gene and its protein product, which is a meiosis-specific transcription factor required for proper expression of many “middle” sporulation genes (Chu and Herskowitz 1998; Hepworth *et al.* 1998; Lindgren *et al.* 2000; Tung *et al.* 2000; Pak and Segall 2002; Shubassi *et al.* 2003). These include *CDC5*, whose polo-like protein kinase product is required for pachytene exit, and also upregulates Ndt80 activation in a feedback loop (Sourirajan and Lichten 2008; Acosta *et al.* 2011), and *CLB1*, which encodes a B-type cyclin that is required for progression through meiosis I (Chu and Herskowitz 1998; Carlile and Amon 2008). Another target of the meiotic recombination checkpoint is the Swe1 protein kinase, which is activated to catalyze inhibitory phosphorylation of Cdk1 at tyrosine 19 (Leu and Roeder 1999). Early work in mitotic cells indicated that Swe1-catalyzed Cdk1 phosphorylation regulates the morphogenesis checkpoint (Lew and Reed 1995). However, it is now known that Swe1 is also a component of one of three Mec1-dependent mechanisms that operate in the S phase checkpoint to prevent cell cycle progression into mitosis (Palou *et al.* 2015).

In our previous studies, we found that deletion of *DMC1* blocks the DNA rereplication induced by Sic1 stabilization (Sawarynski *et al.* 2009). In this report, we describe our further genetic investigation into constituents of the meiotic recombination checkpoint as they pertain to Sic1-induced DNA rereplication. We found that certain upstream components, including *MEC1*, were required to prevent DNA rereplication. However, we did not find evidence that particular downstream effectors that regulate meiotic progression were involved. We further examined processes that operate to prevent DNA replication in the mitotic cell cycle, and found overlap with respect to specific phosphorylation events, including those that are important for blocking late DNA replication origin firing in an S phase checkpoint response. Interestingly, these data suggest a pathway in which the effectors are phosphorylated through a Rad53-independent mechanism.

## Materials and Methods

### Strains

Yeast strains used in this study are listed in Table 1. Construction of the *HOP1pr-SIC1*∆*P^HA^* module, and its integration into the genome, were described previously (Sawarynski *et al.* 2009). In most cases, deletion mutations were generated in haploids by homology-directed site-specific replacement with selectable markers (Baudin *et al.* 1993). These markers were PCR-amplified from either genomic DNA of a deletion set mutant (Winzeler *et al.* 1999) (GE Dharmacon), or from a plasmid (Brachmann *et al.* 1998). Certain mutant progenitor strains in the W303 background were generously provided by other investigators: SKY2939 (*h2a-S129A*) (Downs *et al.* 2004) by Stephen Kron (University of Chicago), YFL234 (*dot1*Δ) (Giannattasio *et al.* 2005) by Marco Muzi-Falconi (Università degli Studi di Milano), U960 (*rad53*Δ *sml1-1*) (Zhao *et al.* 1998) by Stephen Elledge (Harvard University), and Y2359 and Y2573 (*dbf4-4A*, *sld3-38A*, and *mcm5-bob1*) (Zegerman and Diffley 2010) by Philip Zegerman (The Gurdon Institute, UK) and John Diffley (The Francis Crick Institute, UK). These mutations were then introduced into our cell system through crossing. Strain construction generally involved introduction of mutations into *MAT***a** cells and into *MAT*α cells with the *HOP1pr-SIC1*∆*P^HA^* module either present or subsequently added, followed by mating of the two cell types. (Note that the shorthand designation of *SIC1*∆*P^HA^* used for diploids in the text and figures indicates the presence of a single copy of the *HOP1pr-SIC1*∆*P^HA^* element, while other mutant allele designations indicate alteration of both gene copies.) All deletion mutations generated for this study were verified by PCR, and deletion of *SWE1* was further confirmed by western blotting using antibody kindly provided by Doug Kellogg (University of California, Santa Cruz) (Sreenivasan and Kellogg 1999). DNA sequencing was used to validate the presence of certain point mutations in our strains. Epitope tagging of Sic1 (*SIC1^13MYC^*) was performed as described (Longtine *et al.* 1998) in *MAT***a** and *MAT*α cells, which were then mated to generate the diploid. An additional manipulation included 5-fluoro-orotic acid-mediated counter-selection (Boeke *et al.* 1984) to isolate a *rad53*Δ *sml1-1* diploid from a strain containing *HOP1pr-SIC1*Δ*P^HA^*.

**Table 1:** Yeast strains

Strain	Relevant Genotype	Designation
Diploids		
YGB495	*ura3-1/ura3-1*::*HOP1pr-SIC1*∆*P^HA^-URA3*	*SIC1*∆*P^HA^* *^a^*
YGB535	*ura3-1/ura3-1*::*HOP1pr-SIC1*∆*P^HA^-URA3 dmc1*∆::*kanMX4/”*	*SIC1*∆*P^HA^ dmc1*∆*^a^*^,^*^b^*
YGB604	*ura3-1/ura3-1*::*HOP1pr-SIC1*∆*P^HA^-URA3 dmc1*∆::*natR/”*	*SIC1*∆*P^HA^ dmc1*∆*^a^*
YGB679	*ura3-1/ura3-1*::*HOP1pr-SIC1*∆*P^HA^-URA3 dmc1*∆::*natR/” mek1*∆::*kanMX4/”*	*SIC1*∆*P^HA^ dmc1*∆ *mek1*∆
YGB687	*swe1*∆::*kanMX4/”*	*swe1*∆
YGB689	*ura3-1/ura3-1*::*HOP1pr-SIC1*∆*P^HA^-URA3 swe1*∆::*kanMX4/”*	*SIC1*∆*P^HA^ swe1*∆
YGB697	*ura3-1/ura3-1*::*HOP1pr-SIC1*∆*P^HA^-URA3 dmc1*∆::*natR/” swe1*∆::*kanMX4/”*	*SIC1*∆*P^HA^ dmc1*∆ *swe1*∆
YGB700	*ura3-1/ura3-1*::*HOP1pr-SIC1*∆*P^HA^-URA3 dmc1*∆::*natR/” pch2*∆::*kanMX4/”*	*SIC1*∆*P^HA^ dmc1*∆ *pch2*∆
YGB703	*ura3-1/ura3-1*::*HOP1pr-SIC1*∆*P^HA^-URA3 pch2*∆::*kanMX4/”*	*SIC1*∆*P^HA^ pch2*∆
YGB712	*ura3-1/ura3-1*::*HOP1pr-SIC1*∆*P^HA^-URA3 hop1*∆::*kanMX4/”*	*SIC1*∆*P^HA^ hop1*∆
YGB713	*ura3-1/ura3-1*::*HOP1pr-SIC1*∆*P^HA^-URA3 /” dmc1*∆::*natR/” hop1*∆::*kanMX4/”*	*SIC1*∆*P^HA^ dmc1*∆ *hop1*∆
YGB721	*ura3-1/ura3-1*::*HOP1pr-SIC1*∆*P^HA^-URA3 red1*∆::*kanMX4/”*	*SIC1*∆*P^HA^ red1*∆
YGB722	*ura3-1/ura3-1*::*HOP1pr-SIC1*∆*P^HA^-URA3 dmc1*∆::*natR/” red1*∆::*kanMX4/”*	*SIC1*∆*P^HA^ dmc1*∆ *red1*∆
YGB758	*ura3-1/ura3-1*::*HOP1pr-SIC1*∆*P^HA^-URA3 rad9*∆::*kanMX4/”*	*SIC1*∆*P^HA^ rad9*∆
YGB759	*ura3-1/ura3-1*::*HOP1pr-SIC1*∆*P^HA^-URA3 dmc1*∆::*natR/”rad9*∆::*kanMX4/”*	*SIC1*∆*P^HA^ dmc1*∆ *rad9*∆
YGB760	*ura3-1/ura3-1*::*HOP1pr-SIC1*∆*P^HA^-URA3 rad53*∆::*HIS3/” sml1-1/”*	*SIC1*∆*P^HA^ rad53*∆ *sml1-1*
YGB761	*ura3-1/ura3-1*::*HOP1pr-SIC1*∆*P^HA^-URA3 dmc1*∆::*natR/” rad53*∆::*HIS3/” sml1-1/”*	*SIC1*∆*P^HA^ dmc1*∆ *rad53*∆ *sml1-1*
YGB785	*ura3-1/ura3-1*::*HOP1pr-SIC1*∆*P^HA^-URA3 sum1*∆::*kanMX4/”*	*SIC1*∆*P^HA^ sum1*∆
YGB786	*ura3-1/ura3-1*::*HOP1pr-SIC1*∆*P^HA^-URA3 dmc1*∆::*natR/” sum1*∆::*kanMX4/”*	*SIC1*∆*P^HA^ dmc1*∆ *sum1*∆
YGB788	*ura3-1/ura3-1*::*HOP1pr-SIC1*∆*P^HA^-URA3 mec1*∆::*LEU2/” sml1*∆::*kanMX4/”*	*SIC1*∆*P^HA^ mec1*∆ *sml1*∆
YGB789	*ura3-1/ura3-1*::*HOP1pr-SIC1*∆*P^HA^-URA3 dmc1*∆::*natR/” mec1*∆::*LEU2/” sml1*∆::*kanMX4/”*	*SIC1*∆*P^HA^ dmc1*∆ *mec1*∆ *sml1*∆
YGB807	*SIC1^13MYC^*::*kanMX6/”*	*SIC1^13MYC^*
YGB808	*ura3-1/ura3-1*::*HOP1pr-SIC1*∆*P^HA^-URA3 SIC1^13MYC^*::*kanMX6/”*	*SIC1*∆*P^HA^ SIC1^13MYC^*
YGB809	*ura3-1/ura3-1*::*HOP1pr-SIC1*∆*P^HA^-URA3 SIC1^13MYC^*::*kanMX6/” dmc1*∆::*natR/”*	*SIC1*∆*P^HA^ SIC1^13MYC^ dmc1*∆
YGB814	*rad53*∆::*HIS3/” sml1-1/”*	*rad53*∆ *sml1-1**^c^*
YGB866	*ura3-1/ura3-1*::*HOP1pr-SIC1*∆*P^HA^-URA3 mcm5-bob1*::*HIS3/” sld3-38A-10his-13myc*::*kanMX/”*	*SIC1*∆*P^HA^ mcm5-bob1 sld3-38A*
YGB867	*ura3-1/ura3-1*::*HOP1pr-SIC1*∆*P^HA^-URA3 dmc1*∆::*natR/” mcm5-bob1*::*HIS3/” sld3-38A-10his-13myc*::*kanMX/”*	*SIC1*∆*P^HA^ dmc1*∆ *mcm5-bob1 sld3-38A*
YGB934	*ura3-1/ura3-1*::*HOP1pr-SIC1*∆*P^HA^-URA3 dmc1*∆::*natR/” hta1-S129A*::*his3MX6/” hta2-S129A*::*TRP1/”*	*SIC1*∆*P^HA^ dmc1*∆ *h2a-S129A*
YGB938	*ura3-1/ura3-1*::*HOP1pr-SIC1*∆*P^HA^-URA3 dmc1*∆::*natR/” mek1*∆::*kanMX4/” rad54*∆::*TRP1/rad54*∆::*HIS3*	*SIC1*∆*P^HA^ dmc1*∆ *mek1*∆ *rad54*∆
YGB966	*ura3-1/ura3-1*::*HOP1pr-SIC1*∆*P^HA^-URA3 dmc1*∆::*natR/” dot1*∆::*kanMX6/”*	*SIC1*∆*P^HA^ dmc1*∆ *dot1*∆
YGB967	*ura3-1/ura3-1*::*HOP1pr-SIC1*∆*P^HA^-URA3 dmc1*∆::*natR/” hta1-S129A*::*his3MX6/” hta2-S129A*::*TRP1/” dot1*∆::*kanMX6/”*	*SIC1*∆*P^HA^ dmc1*∆ *h2a-S129A dot1*∆
YGB1012	*ura3-1/ura3-1*::*HOP1pr-SIC1*∆*P^HA^-URA3 rad54*∆::*TRP1/rad54*∆::*HIS3*	*SIC1*∆*P^HA^ rad54*∆
YGB1014	*ura3-1/ura3-1*::*HOP1pr-SIC1*∆*P^HA^-URA3 hta1-S129A*::*his3MX6/” hta2-S129A*::*TRP1/”*	*SIC1*∆*P^HA^ h2a-S129A*
YGB1075	*ura3-1/ura3-1*::*HOP1pr-SIC1*∆*P^HA^-URA3 dmc1*∆::*natR/” dbf4*∆::*TRP1/” his3*::*P_DBF4_-dbf4-4A*::*HIS3/” sld3-38A-10his-13myc*::*kanMX/”*	*SIC1*∆*P^HA^ dmc1*∆ *dbf4-4A sld3-38A*
YGB1241	*ura3-1/ura3-1*::*HOP1pr-SIC1*∆*P^HA^-URA3 dmc1*∆::*natR/” sml1*∆::*kanMX4/”*	*SIC1*∆*P^HA^ dmc1*∆ *sml1*∆
YGB1255	*ura3-1/ura3-1*::*HOP1pr-SIC1*∆*P^HA^-URA3 DMC1/dmc1*∆::*natR dbf4*∆::*TRP1/” his3*::*P_DBF4_-dbf4-4A*::*HIS3/” sld3-38A-10his-13myc*::*kanMX/”*	*SIC1*∆*P^HA^ dbf4-4A sld3-38A*
Haploid		
YGB502	*SIC1^13MYC^*::*kanMX6*	*SIC1^13MYC^*

All strains listed were constructed in the W303 background (Thomas and Rothstein 1989): diploid wild type = *MAT***a***/*α *ade2-1/” ura3-1/” leu2-3,112/” his3-11,15/” trp1-1/” can1-100/*”; haploid wild type = *MAT***a** *ade2-1 ura3-1 leu2-3,112 his3-11,15 trp1-1 can1-100*.

a Sawarynski *et al.* (2009).

b This *SIC1*∆*P^HA^ dmc1*∆ strain was used only for the experiment shown in Figure S2.

c Derived from YGB760.

### Cell culture

All yeast incubations were conducted at 30°. Meiosis was induced by starvation based on an established procedure for synchronous sporulation (Padmore *et al.* 1991). In this method, yeast cells were first grown on solid [2% (w/v) agar] YPG medium [1% (w/v) yeast extract, 2% (w/v) peptone, 3% (v/v) glycerol], or, alternatively, on solid YPD medium [1% (w/v) yeast extract, 2% (w/v) peptone, 2% (w/v) dextrose] for 3–4 d, and single colonies were used to inoculate YPD liquid cultures. The overnight YPD cultures were then used to inoculate YPA [1% (w/v) yeast extract, 2% (w/v) peptone, 2% (w/v) potassium acetate] at an OD_600_ of ∼0.2. Cells were incubated overnight, typically for 16 hr, and then resuspended at equivalent cell densities (based on OD_600_ values) for strains within an experiment in sporulation medium consisting of 0.3% (w/v) potassium acetate and 0.02% (w/v) raffinose supplemented with leucine, arginine, and histidine each at 250 μM, tryptophan at 100 μM, and uracil at 50 μM. Cells were returned to incubation at time 0, and aliquots were harvested at indicated time points for flow cytometry and protein analyses (see below). For most experiments, control strains *SIC1*∆*P^HA^* and *SIC1*∆*P^HA^dmc1*∆ were included. Each experimental strain was analyzed at least twice in independent experiments, and in many cases more than twice (see Supplemental Material, Figure S1).

To conduct a synchronized mitotic cell cycle timecourse, *MAT***a** cells grown to saturation in YPD were brought to an OD_600_ of ∼0.2 and incubated for 2 hr. The yeast mating pheromone α-factor (Zymo Research) was then added to a final concentration of 2.5 μM, and cells were incubated for an additional 2 hr to achieve G1 arrest. The cells were then washed with sterile water to remove α-factor, resuspended in fresh YPD, and further incubated. Aliquots were taken at 15-min intervals for flow cytometry and western blotting analyses (see below). For examining the response to inhibition of DNA replication, cells were arrested with α-factor as described above and then released into 0.8× YPD containing 0.2 M HU (MP Biomedicals).

### DNA content

Cells were harvested by centrifugation, resuspended in 70% ethanol and stored at 4°. Aliquots of the fixed cells were washed once with 50 mM Tris-HCl, pH 7.5, resuspended in 1 ml of the same buffer, and then treated with 250 μg RNase A for 1 hr at 37°, followed by 250 μg proteinase K for 1 hr at 37°. Digested samples were incubated with 10× SYBR Green I (Molecular Probes) at 4° overnight, sonicated briefly, and analyzed with a FACSCanto II flow cytometer (BD Biosciences) (or, in one experiment, a BD LSR II flow cytometer) (Microscopy, Imaging, and Cytometry Resources Core at Wayne State University School of Medicine). DNA content histograms were generated using WinMDI freeware. DNA rereplication was quantified by using the gating function in WinMDI to determine the number of events (out of 20,000) that were recorded with > 4C DNA content. Gating was established based on the 4C DNA peak, and was held constant within each individual experiment.

### Protein

Cells were harvested by centrifugation and stored at −70°. For most experiments, denatured whole-cell extracts were prepared based on an alkaline extraction method (Kushnirov 2000). In the case of Rad53 analysis, a tricholoroacetic acid bead beating method was used, as described (Foiani *et al.* 1994). Resulting samples were subjected to SDS-polyacrylamide electrophoresis. For western blotting, the separated proteins were transferred to nitrocellulose (GE Healthcare). Primary antibodies included rat anti-α-tubulin (Serotec), mouse anti-hemagglutinin (Covance), rabbit anti-yeast γ-H2A (generously provided by Christophe Redon and William Bonner, National Cancer Institute) (Nakamura *et al.* 2006), mouse anti-myc (Santa Cruz), and rabbit anti-Rad53 (Abcam). Signals were generated with IRDye 800-conjugated goat anti-rat (Rockland), Alexa Fluor 680 goat anti-rabbit (Invitrogen), or Alexa Fluor 680 goat anti-mouse (Invitrogen) secondary antibodies. Reactive bands were visualized with an Odyssey infrared imaging system (Li-Cor). For analysis of Rad53 activity, separated proteins were transferred to PVDF (Millipore), and Rad53 autophosphorylation was analyzed *in situ* as described (Foiani *et al.* 1994). In the figures, line borders indicate cropping, and vertically contiguous panels indicate data originating from the same blot or membrane.

### Data availability

The authors state that all data necessary for confirming the conclusions presented in the article are represented fully within the article.

## Results

We have developed a system in which cells undergoing meiosis experience at least one extra round of DNA replication (Sawarynski *et al.* 2009). This phenotype occurs upon deregulation of CDK activity through early meiosis-specific expression (via the *HOP1* promoter) of *SIC1*∆*P^HA^*, encoding a Sic1^HA^ variant lacking critical CDK phosphorylation sites that are necessary for its destruction (Verma *et al.* 1997). DNA rereplication does not occur in this system when the Sic1^HA^ phosphorylation sites are not altered, as this version of the protein is subject to post-translational modification and degradation. Interestingly, deletion of *DMC1* (*dmc1*Δ) activates a response that prevents the DNA rereplication phenotype normally observed in our specially engineered cells. We have examined this pathway genetically, as described below. The extent of DNA rereplication in the various strains that we describe in this study was quantified from flow cytometry data, and is presented as a compilation in Figure S1.

### MEC1

**Figure 1:**
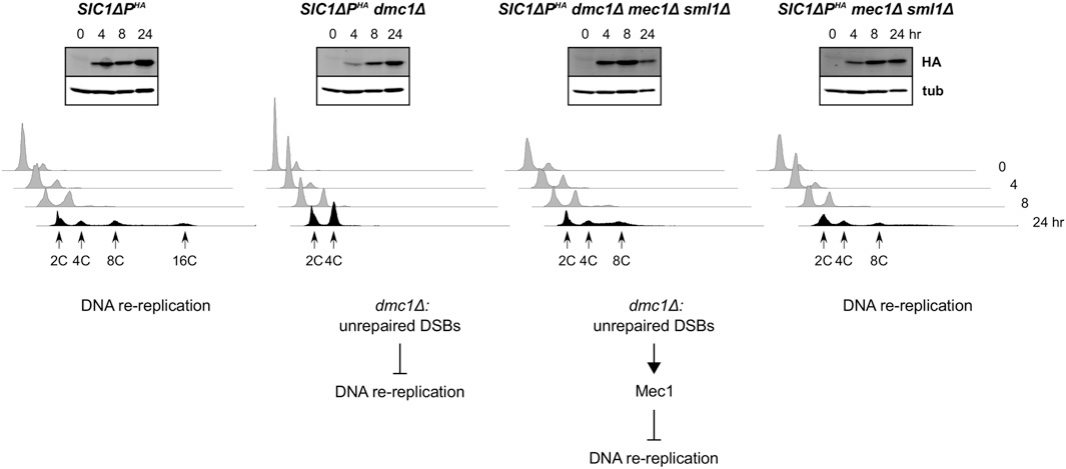
*MEC1* is required for *dmc1*Δ-dependent inhibition of *SIC1*∆*P^HA^*-induced DNA rereplication. Strains were treated to enter the meiotic program in a synchronous fashion. At the indicated time points, samples were analyzed for Sic1ΔP^HA^ (HA) and the tubulin control (tub) by western blotting (upper panels), and for DNA content by flow cytometry (lower histograms). For this figure, and others that follow, the 4C designation indicates the population of cells that have undergone one round of DNA replication; cells with rereplicated DNA appear to the right of this position, with peaks of cells and approximate DNA contents >4C indicated. Conclusions for this experiment are provided as schematics to provide an example of pathway analysis.

Previously, we showed that *RAD17*, which encodes a 9-1-1 member, is required to suppress *dmc1*Δ-dependent inhibition of DNA rereplication, suggesting that a branch of the meiotic recombination checkpoint could affect DNA replication (Sawarynski *et al.* 2009). Given that Rad17 is intimately associated with Mec1, we suspected that this central protein kinase was also involved. To test this hypothesis, we generated a *mec1* null mutant in our strain background. Because *MEC1* is essential for viability (Kato and Ogawa 1994), it was necessary to delete *SML1* as well to suppress the lethality resulting from *MEC1* loss (Zhao *et al.* 1998). As shown in Figure 1, DNA rereplication was observed in *SIC1*∆*P^HA^dmc1*∆ *mec1*∆ *sml1*∆ cells. This phenotype was not due to *sml1*∆, as *SIC1*∆*P^HA^dmc1*∆ *sml1*∆ cells did not exhibit DNA rereplication (see Figure S1). Therefore, *MEC1* was involved in the prevention of DNA rereplication in *SIC1*∆*P^HA^dmc1*∆ cells. We observed less robust DNA rereplication in *SIC1*∆*P^HA^dmc1*∆ *mec1*∆ *sml1*∆ cells than in *SIC1*∆*P^HA^* cells; a portion of this effect may have been due to the absence of *MEC1* and *SML1* as revealed by examination of *SIC1*∆*P^HA^mec1*∆ *sml1*∆ cells (Figure 1 and Figure S1).

### MEK1 and associated genes

**Figure 2:**
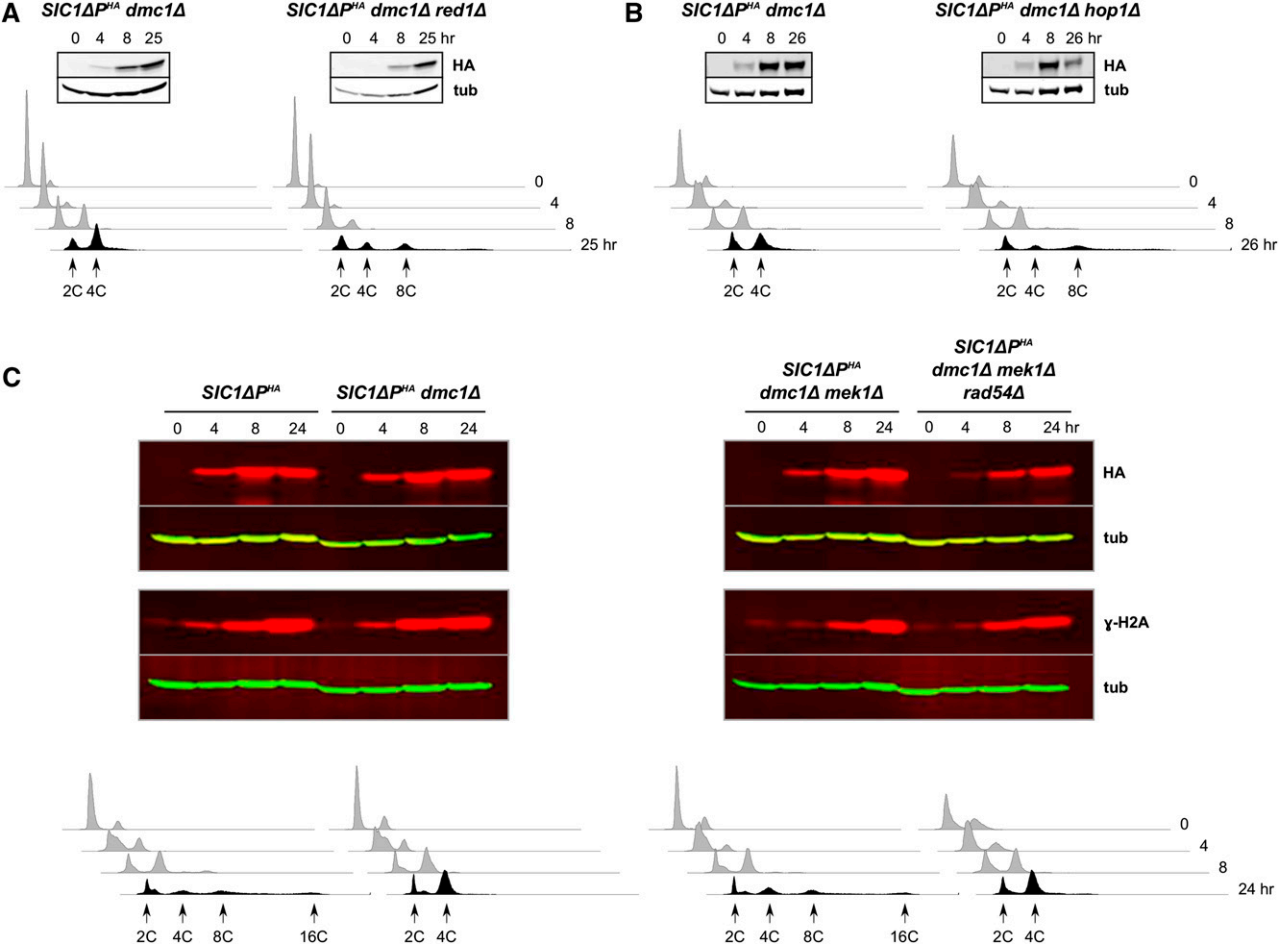
Factors that enforce interhomolog bias and prevent meiotic progression are required for *dmc1*Δ-dependent inhibition of *SIC1*∆*P^HA^*-induced DNA rereplication. Cells were treated to enter meiosis and analyzed for protein levels by western blotting, and for DNA content by flow cytometry. (A, B), the effect of *red1*∆ and *hop1*∆, respectively; (C), the effect of *mek1*∆, as well as *rad54*∆. Western blotting analysis included Sic1ΔP^HA^ (HA), tubulin (tub) and phosphorylated H2A (γ-H2A).

We further examined genes that operate downstream of *MEC1* in the established response that prevents both intersister repair and meiotic progression, including those that encode the axial proteins Mek1, Red1, and Hop1. As in the case of *mec1*∆, deletion of any one of these genes restored DNA rereplication in *SIC1*∆*P^HA^dmc1*Δ cells (Figure 2), indicating that they were required for prevention of DNA rereplication in response to the accumulation of unrepaired DSBs. In these cases, DNA rereplication was robust, as exhibited by the generation of cells with high DNA content in some cases reaching ∼16C (Figure 2 and Figure S1).

It has been shown that *dmc1*∆ *mek1*∆ cells that are also *rad54*∆, and therefore incapable of completing intersister repair (Arbel *et al.* 1999) progress through meiosis, *albeit* with slower kinetics than wild-type or *dmc1*Δ *mek1*Δ cells (Cartagena-Lirola *et al.* 2008; Chuang *et al.* 2012); this phenotype illustrates the checkpoint function of *MEK1*. To determine whether the *MEK1* function to suppress intersister repair, or to prevent meiotic progression, was at play in our specialized case, we examined *SIC1*∆*P^HA^dmc1*∆ *mek1*∆ *rad54*∆ cells. We found that the recovery of DNA rereplication observed in *SIC1*∆*P^HA^dmc1*Δ *mek1*Δ cells was not observed with the addition of *rad54*∆ (Figure 2C). In each strain, we observed an increase in phosphorylated histone H2A (γ-H2A), which is generated through Mec1 (and related Tel1) catalysis in response to DSB formation, and leads to extensive regions of chromatin containing γ-H2A on either side of the DSB (Shroff *et al.* 2004). These data suggest a persistence of DSBs in our cells throughout the time course, regardless of *DMC1* or *RAD54* status. We further demonstrated that *RAD54* itself was not required for the DNA rereplication phenotype (Figure S2). These data indicate that *MEK1* inhibited DNA rereplication by preventing intersister repair and maintaining the DSB-induced signal rather than by influencing DNA replication itself.

Studies have indicated that the AAA+-type ATPase Pch2 suppresses intersister repair to some extent, and helps to prevent meiotic progression when unrepaired DSBs accumulate (San-Segundo and Roeder 1999; Ho and Burgess 2011; Zanders *et al.* 2011; Chen *et al.* 2014). We found that deletion of *PCH2* relieved the *dmc1*∆-dependent inhibition of DNA rereplication in our *SIC1*∆*P^HA^* system (Figure S3), indicating that *PCH2* aided in preventing DNA rereplication. In this case, as in certain other mutants analyzed (see below), few cells with DNA content >∼8C were observed. Extensive DNA rereplication was detected with *SIC1*∆*P^HA^pch2*∆ cells (Figure S1 and Figure S3), indicating that *PCH2* itself was not required for the DNA rereplication phenotype in *SIC1*∆*P^HA^* cells.

### CDK

**Figure 3:**
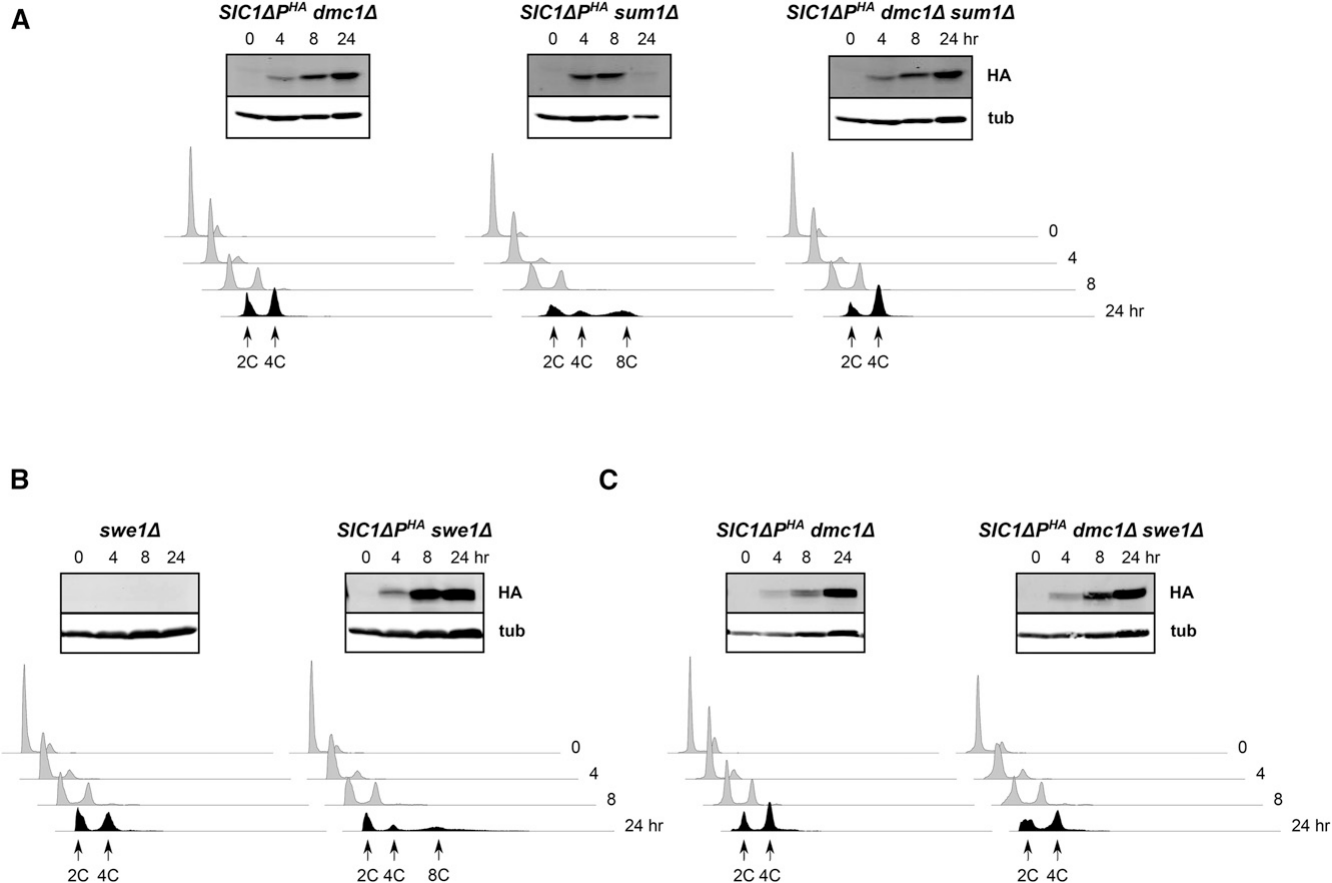
Regulators of Cdk1 activation are not required for *dmc1*Δ-dependent inhibition of DNA rereplication. Cells were treated to enter meiosis and examined for Sic1ΔP^HA^ (HA) and tubulin (tub) by western blotting, and for DNA content by flow cytometry. (A) the effect of *sum1*∆; (B, C), the effect of *swe1*∆. Note that the *SIC1*Δ*P^HA^ dmc1*Δ control in (A) is identical to that shown in Figure 1.

We considered the possibility that the checkpoint response might prevent CDK activation to inhibit DNA rereplication. One mechanism by which the meiotic recombination checkpoint prevents meiotic progression is through Sum1, a key transcription factor that represses expression of middle sporulation genes normally induced by the transcription factor Ndt80 (Lindgren *et al.* 2000; Pak and Segall 2002; Winter 2012). Included among these Ndt80-induced genes are those that encode the B-type cyclins Clb1, -3, -4, -5, and -6 (Chu and Herskowitz 1998). We found that DNA rereplication occurred in *SIC1*∆*P^HA^sum1*Δ cells, although with reduced efficiency for cells with DNA content >∼8C, but not in *SIC1*∆*P^HA^dmc1*Δ *sum1*Δ cells (Figure 3A and Figure S1), indicating that *SUM1* was not involved in this checkpoint.

We also examined *SWE1*, whose product becomes activated in the meiotic recombination checkpoint response to catalyze inhibitory phosphorylation of Cdk1 at tyrosine 19 (Leu and Roeder 1999). We found that deletion of *SWE1*, like that of *SUM1*, did not prevent DNA rereplication in *SIC1*∆*P^HA^* cells, nor did it reverse the *dmc1*Δ-dependent inhibition of DNA rereplication (Figure 3, B and C), suggesting that *SWE1* was not required for prevention of DNA rereplication. It is noted that because Ndt80 levels and activity are downregulated by the meiotic recombination checkpoint response, thereby lowering B-type cyclin availability and CDK activity, we might not expect to see much of an effect by simply deleting *SWE1* in our cells. However, *dmc1*∆ *swe1*∆ cells do progress into MI, although with delayed kinetics relative to wild type cells (Leu and Roeder 1999). During the course of these studies, we also examined a *swe1*Δ mutant without the *SIC1*∆*P^HA^* allele. This experiment was prompted by the report that *swe1*Δ cells rereplicate their DNA during meiosis, a phenotype that is different from ours in that multispore asci are formed (Rice *et al.* 2005). We did not observe these phenotypes in our *swe1*Δ cells, perhaps due to differences in strain types or culture conditions (Figure 3B and data not shown).

**Figure 4:**
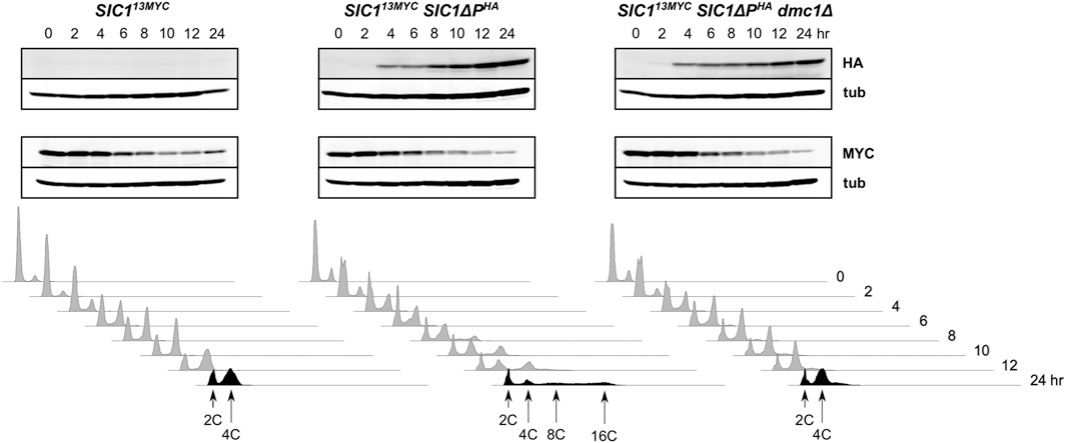
The CDK inhibitor Sic1 does not accumulate in *SIC1*∆*P^HA^ dmc1*Δ cells. The indicated cells containing Sic1^13MYC^ were treated to enter meiosis, and then analyzed at several time points for Sic1ΔP^HA^ (HA), Sic1^13MYC^ (MYC) and tubulin (tub) by western blotting, and for DNA content by flow cytometry.

To investigate another CDK regulator, Sic1, we generated strains in which Sic1 was tagged with MYC epitope repeats. In this way, we could distinguish endogenous Sic1 from the induced version lacking CDK-targeted phosphorylation sites, which is tagged with the HA epitope. We first examined a haploid strain during the cell cycle to ensure that our Sic1^13MYC^ protein behaved properly. As expected, we found that Sic1 disappeared almost completely as cells progressed from G1 arrest, and then reappeared after S phase was completed (Figure S4). We next examined diploid strains in meiosis (Figure 4). Synchrony is difficult to achieve in meiosis, and we did not observe the sharp reduction and reappearance of Sic1 in our wild-type cells. However, we did see a decline in Sic1 as cells progressed through the time course, which may have reflected a real decrease in cellular Sic1 steady state levels. As shown, we observed nearly identical Sic1^13MYC^ profiles in *SIC1*∆*P^HA^* and *SIC1*∆*P^HA^dmc1*Δ cells. We conclude that a decrease in CDK activity through stabilization of endogenous Sic1 was not likely to be responsible for *dmc1*Δ-dependent inhibition of DNA rereplication.

### γ-H2A and DOT1

**Figure 5:**
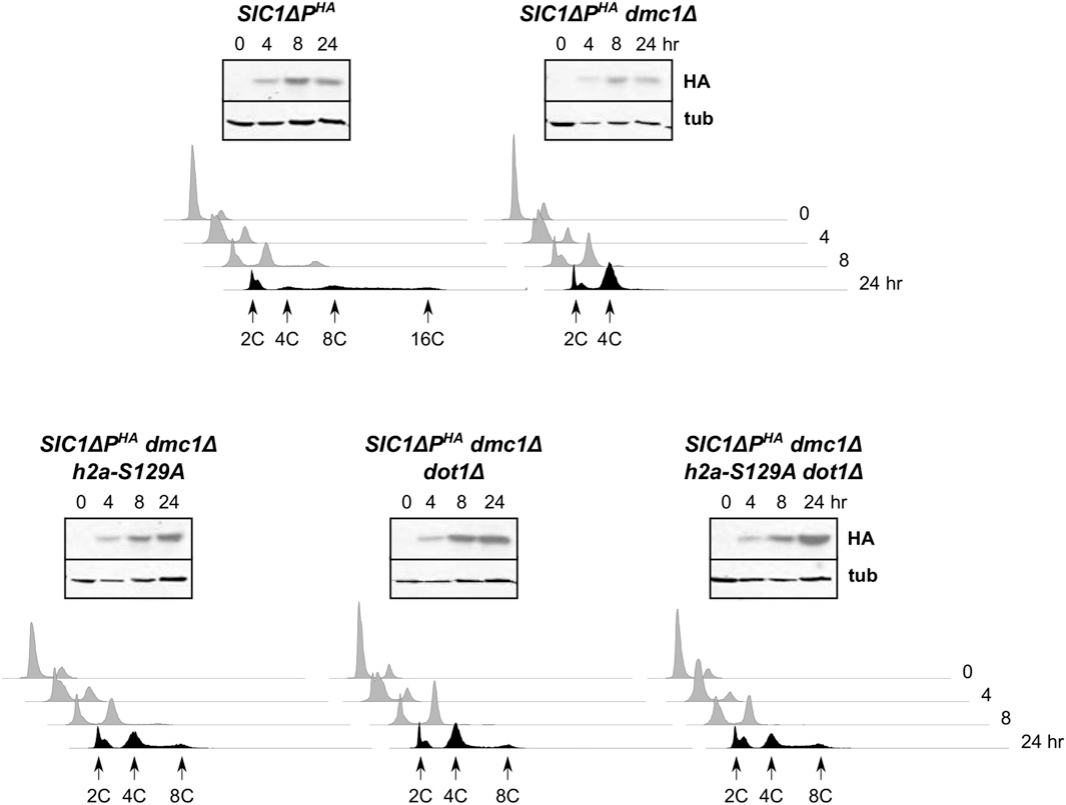
γ-H2A and *DOT1* are involved in *dmc1*Δ-dependent inhibition of *SIC1*∆*P^HA^*-induced DNA rereplication. The indicated strains were treated to enter meiosis and then analyzed for Sic1ΔP^HA^ (HA) and tubulin (tub) by western blotting, and for DNA content by flow cytometry.

Mec1- and Tel1-catalyzed H2A phosphorylation at serine 129, which generates γ-H2A, is thought to be an important protective mechanism that promotes DSB repair to prevent genomic alterations (Downs *et al.* 2000; Redon *et al.* 2003), and functions in the G1 DNA damage checkpoint during the mitotic cell cycle (Javaheri *et al.* 2006; Hammet *et al.* 2007). Because *MEC1* was required in the meiotic recombination checkpoint response that prevents DNA rereplication, we suspected that one of its major targets would be involved as well. We found that γ-H2A was generated in *H2A* (*HTA1* and *HTA2*) cells regardless of *DMC1* status (see Figure 2C). We generated a *SIC1*Δ*P^HA^* strain with *HTA1* and *HTA2* both mutated (*h2a-S129A*) so that the two H2A subunits could not be phosphorylated at serine 129, and confirmed through western blotting that these cells were devoid of γ-H2A (see Figure S2 and Figure S5). Importantly, absence of γ-H2A led to DNA rereplication in *SIC1*∆*P^HA^dmc1*Δ cells; while not as extensive as in *SIC1*∆*P^HA^* cells with regard to total number of cells exhibiting >4C DNA content and those reaching >∼8C, the DNA rereplication was obvious, and observed consistently (Figure 5 and Figure S1). *SIC1*∆*P^HA^ h2a-S129A* cells rereplicated their DNA with clear evidence of cells containing >∼8C DNA content (Figure S2).

We also examined the phenotype resulting from deletion of *DOT1*, which encodes a histone methyltransferase required for the meiotic recombination checkpoint response (San-Segundo and Roeder 2000). Dot1 catalyzes methylation of histone H3 at lysine K79 (H3meK79) (Lacoste *et al.* 2002; Ng *et al.* 2002; van Leeuwen *et al.* 2002), and, like γ-H2A, is important for the G1 DNA damage checkpoint in the mitotic cell cycle (Giannattasio *et al.* 2005; Wysocki *et al.* 2005). Similar to the case with *h2a-S129A*, we observed a modest degree of DNA rereplication in *SIC1*∆*P^HA^dmc1*Δ *dot1*∆ cells, and combination of the *dot1*∆ and *h2a-S129A* mutations did not enhance this effect (Figure 5 and Figure S1). These data suggest that γ-H2A and Dot1 operated in the same pathway in preventing DNA rereplication in *SIC1*∆*P^HA^dmc1*Δ cells.

### RAD53

**Figure 6:**
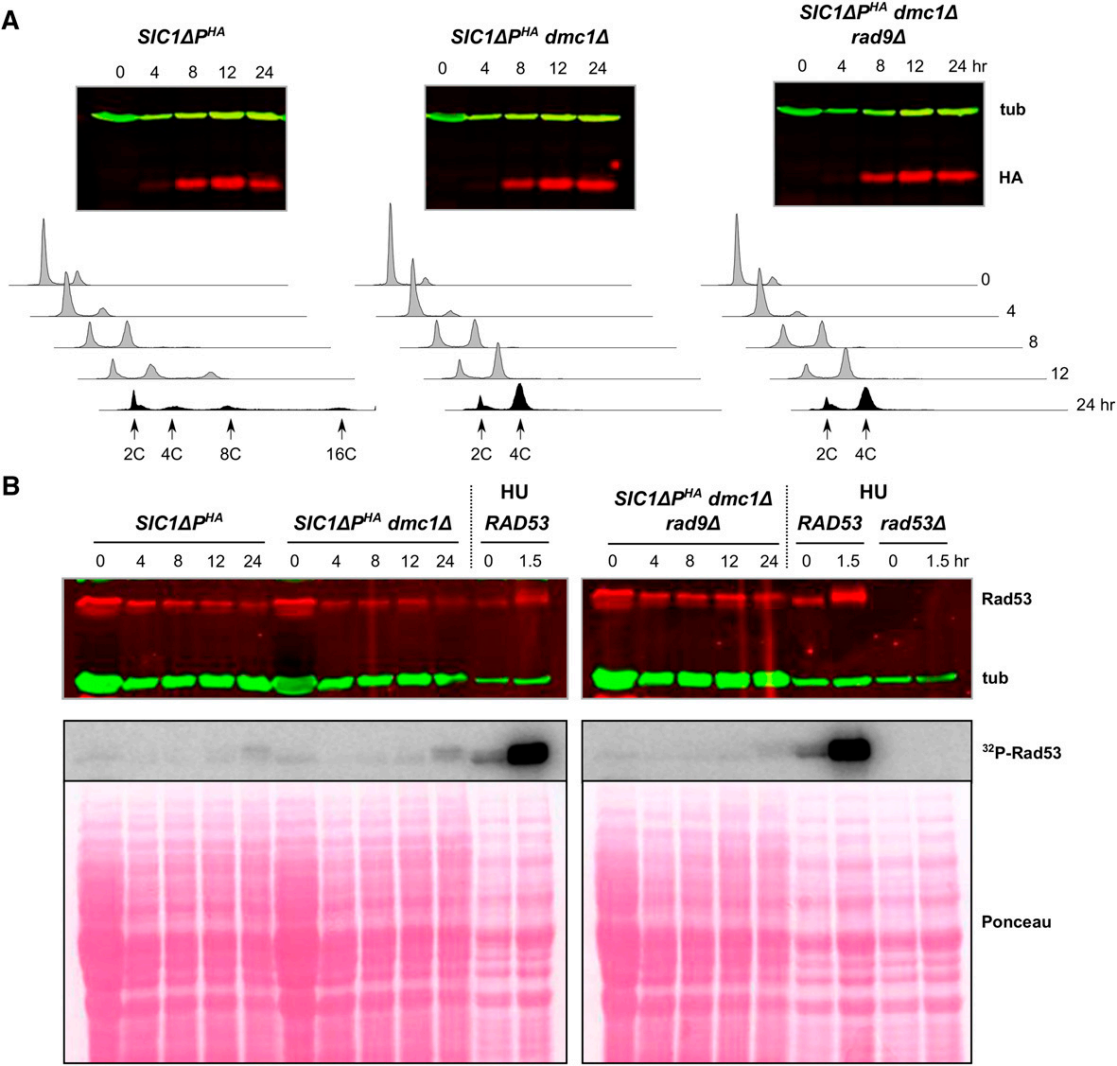
Rad53 is not activated by deletion of *DMC1*. (A) The indicated strains were treated to enter meiosis and then analyzed for: Sic1ΔP^HA^ (HA) and tubulin (tub) by western blotting, and for DNA content by flow cytometry. (B) Samples from the same time course shown in (A) were used to assess Rad53 activation through western blotting [Rad53 and tubulin (tub) top panel], and Rad53 autophosphorylation *in situ* (^32^P-Rad53, middle panel). Total protein loading for the autophosphorylation assay was determined by Ponceau S staining (bottom panel). Control samples included vegetative *SIC1*Δ*P^HA^ dmc1*Δ (*RAD53*) and *rad53*Δ *sml1-1* (*rad53*Δ) diploids exposed to HU for the times indicated.

While Rad53 is not involved in the meiotic recombination checkpoint *per se*, it can be activated by genotoxic stress during meiosis (Weber and Byers 1992; Cartagena-Lirola *et al.* 2008; Blitzblau and Hochwagen 2013). Therefore, we elected to determine whether or not *RAD53* was involved in the checkpoint that prevents DNA rereplication in our system. (As in the case of *mec1*Δ, *rad53*Δ cells are viable when *SML1* is also defective (Zhao *et al.* 1998)). We were surprised to find that DNA rereplication did not occur in *SIC1*∆*P^HA^rad53*∆ *sml1-1* cells, regardless of *DMC1* status (Figure S6), even after 48 hr (data not shown). As an alternative genetic test, we turned to *RAD9*, which encodes a protein that mediates Rad53 activation in many circumstances (Sun *et al.* 1998; Vialard *et al.* 1998; Gilbert *et al.* 2001). By contrast to *rad53*Δ, *rad9*∆ did not prevent DNA rereplication in *SIC1*∆*P^HA^* cells (Figure S6). Importantly, *RAD9* was not required for suppressing DNA rereplication in *SIC1*∆*P^HA^dmc1*Δ cells (Figure 6A), indicating that *RAD9* was not involved in this checkpoint response.

**Figure 7:**
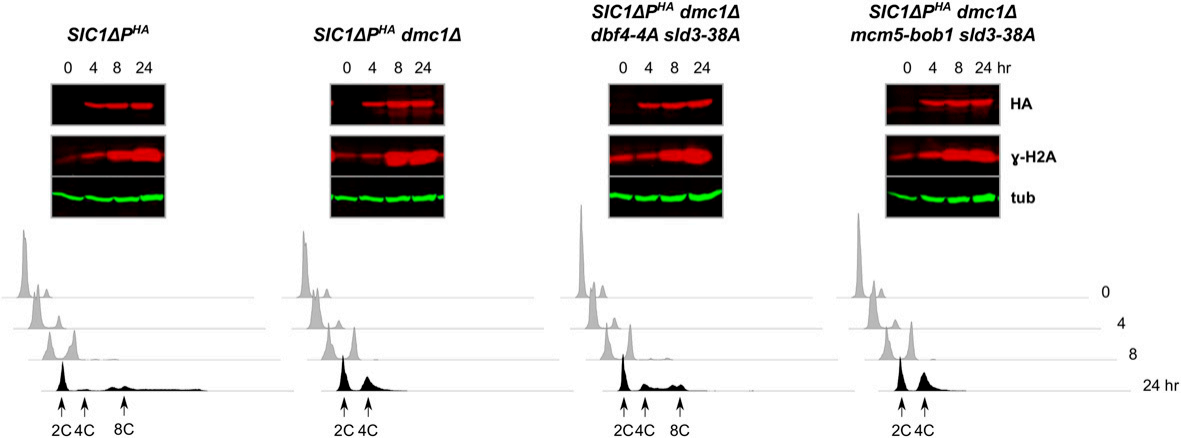
Sites of phosphorylation targeted by Rad53 in the mitotic cell cycle influence *dmc1*Δ-dependent inhibition of *SIC1*∆*P^HA^*-induced DNA rereplication. The indicated strains were treated to enter meiosis, and then analyzed for Sic1ΔP^HA^ (HA) and tubulin (tub) by western blotting and DNA content by flow cytometry.

Because our results precluded analysis of Rad53 function through gene deletion, we explored the possibility of a Rad53, or Rad53-like, function through different means. In response to replication fork stalling or DNA damage during S phase of the mitotic cell cycle, Rad53 is activated to catalyze phosphorylation of Dbf4 and Sld3, thereby preventing firing of late origins (Lopez-Mosqueda *et al.* 2010; Zegerman and Diffley 2010). We examined two different types of mutants to determine whether this process could be involved in our system. The first involved cells containing mutant alleles of both *DBF4* and *SLD3*, encoding proteins with alterations in important Rad53-targeted phosphorylation sites. *SIC1*∆*P^HA^dmc1*∆ cells containing the *dbf4-4A* and *sld3-38A* alleles exhibited DNA rereplication, indicating that phosphorylation of Dbf4 or Sld3, or both proteins, was required for full prevention of DNA rereplication (Figure 7 and Figure S1). We noticed that, as in the case of *SIC1*∆*P^HA^dmc1*∆ *h2A-S129A* cells, these cells appeared to rereplicate their DNA less than *SIC1*∆*P^HA^* cells, in that they did not exhibit >∼8C DNA content. In *SIC1*∆*P^HA^ dbf*4-*4A sld3-38A* cells, we did observe cells with >∼8C DNA content, indicating that the phosphorylation site mutations themselves were not responsible for limiting DNA rereplication (Figure S7). The second type of mutant cells contained the *sld3-38A* allele and *mcm5-bob1*, a mutant allele that bypasses the essential function of *DBF4* (Hardy *et al.* 1997). DNA rereplication was not observed in *SIC1*∆*P^HA^dmc1*∆ *mcm5-bob1 sld3-38A* cells (Figure 7). We confirmed that the *mcm5-bob1* allele did not prevent DNA rereplication in our system (Figure S7). While it might be expected that cells with altered Dbf4 phosphorylation sites would behave similarly to cells with *mcm5-bob1*, as in the mitotic cell cycle studies, certain experimental factors may account for this discrepancy (see *Discussion*). These data suggest that Dbf4 phosphorylation was sufficient to prevent DNA rereplication, at least in the context of the *mcm5-bob1* allele. It is noted that either Dbf4 or Sld3 phosphorylation alone can contribute to prevention of late origin firing in mitotic S phase (Lopez-Mosqueda *et al.* 2010; Zegerman and Diffley 2010). We conclude that sites normally phosphorylated by Rad53 in the mitotic cell cycle also functioned to prevent DNA replication during meiosis under our conditions that promote DNA rereplication.

To further investigate a possible Rad53 function, we examined Rad53 enzymatic activity. While Rad53 isoforms with reduced electrophoretic mobility are indicative of its phosphorylation and activation, a more sensitive method is to analyze Rad53 autophosphorylation *in situ* (Pellicioli *et al.* 1999). As can be seen in Figure 6B, a slight Rad53 activation was observed at the late time point in the *SIC1*Δ*P^HA^*, *SIC1*Δ*P^HA^dmc1*Δ, and *SIC1*Δ*P^HA^dmc1*Δ *rad9*Δ strains. This activation was negligible relative to HU-induced Rad53 activation in mitotic cells. While it appeared that the Rad53 activation was a bit higher in the *SIC1*Δ*P dmc1*Δ cells, it is unlikely that this degree of activation could have been responsible for the checkpoint, given that heightened activation was not observed in the checkpoint-proficient *SIC1*Δ*P^HA^dmc1*Δ *rad9*Δ cells. It is possible that persistence of DSBs and accumulation of DNA damage over time in *SIC1*Δ*P^HA^dmc1*Δ cells led to low level Rad9-dependent Rad53 phosphorylation.

## Discussion

When *S. cerevisiae* cells deleted for *DMC1* are induced to enter the meiotic program, a response is activated that inhibits recombination between sister chromatids and prevents progression into the meiotic divisions (Bishop *et al.* 1992; Xu *et al.* 1997; Wan *et al.* 2004; Niu *et al.* 2005). We have observed that cells undergoing meiosis but engineered to rereplicate their DNA through expression of *SIC1*∆*P^HA^* also respond to deletion of *DMC1* by preventing extra DNA replication (Sawarynski *et al.* 2009). Our previous studies indicated that this response requires *RAD17*, suggesting a checkpoint mechanism. Our further genetic analysis shown here has confirmed that a checkpoint response is involved.

We observed differences in the degree of DNA rereplication recovery in *SIC1*∆*P^HA^dmc1*Δ cells depending on which additional gene was deleted or mutated. While experimental variability makes it difficult to be conclusive about these differences, we observed trends in particular circumstances. For example, removal of genes encoding certain proteins, such as Mek1, that serve to prevent both intersister repair and meiotic progression led to extensive DNA rereplication including cells containing >∼8C DNA content. Our experiments further suggest that the *MEK1* function to prevent intersister repair, and to thereby retain the checkpoint signal originating from unrepaired DSBs, is operative in preventing DNA rereplication in our cells. By contrast to these gene deletions, mutation of genes to abrogate H2A or Dbf4 and Sld3 phosphorylation led to less DNA rereplication, with few cells detected containing >∼8C DNA content. This difference might indicate that the degree of DNA rereplication is limited by the presence of unrepaired and resected DSBs, although we did observe considerable γ-H2A staining in cells, regardless of checkpoint status. In addition, more than a single checkpoint mechanism may be involved in preventing DNA rereplication, with only one being absent in certain cases such as *SIC1*∆*P^HA^dmc1*Δ *dbf4-4A sld3-38A* cells. In this sense, perhaps the checkpoint pathway that we have uncovered affects only a subset of origins. Regardless, the data provide clear evidence that the meiotic recombination checkpoint can target the DNA replication machinery.

The meiotic recombination checkpoint response that prevents DNA rereplication shares several components with cell cycle DNA damage response checkpoint mechanisms. Genes encoding Mec1 and Rad17, and, presumably, the entire 9-1-1 complex that includes Rad17 and facilitates Mec1 function, were required in our system. It is interesting to note that DNA rereplication itself in the mitotic cell cycle induces a checkpoint response dependent on *MEC1* and *RAD17* that restricts the extent of DNA rereplication (Archambault *et al.* 2005). In our case, we observed slightly less DNA rereplication upon deletion of *MEC1* and *SML1* in *SIC1*∆*P^HA^dmc1*Δ cells when compared with *SIC1*∆*P^HA^* cells deleted for genes such as *MEK1*, particularly with regard to >∼8C DNA content cells. (The possibility is noted that deletion of *MEC1* and *SML1* might have had a minor effect on DNA rereplication in *SIC1*∆*P^HA^* cells as well). It has been reported that *dmc1*Δ *mec1-1* cells continue to progress through meiosis with unrepaired DSBs (Lydall *et al.* 1996); as suggested above, the presence of unrepaired DSBs may influence the degree of DNA rereplication in our system. We did not interrogate *TEL1*, which encodes a close relative of Mec1 that is involved in DNA damage response pathways, including the meiotic recombination checkpoint response (Greenwell *et al.* 1995; Morrow *et al.* 1995; Usui *et al.* 2001). Like Mec1, Tel1 catalyzes Hop1 phosphorylation, which is required for Mek1 activation (Carballo *et al.* 2008). It is possible that the phenotypic difference between *SIC1*∆*P^HA^dmc1*Δ *mec1*Δ *sml1*Δ and *SIC1*∆*P^HA^dmc1*Δ *mek1*Δ cells (observed despite the fact that Mek1 functions downstream of Mec1) is due to the presence of Tel1. In addition, there might exist downstream effectors of Mec1, other than Mek1, that have an influence by promoting DNA rereplication.

Mec1 activation involves the interaction of its partner Ddc2 with single-stranded-DNA bound replication protein A (Zou and Elledge 2003), which, in the case of *dmc1*Δ cells would be formed readily due to the highly resected Spo11-generated DSBs (Bishop *et al.* 1992). In turn, Mec1 can catalyze formation of γ-H2A, which we found contributed to prevention of DNA rereplication, as did *DOT1*, encoding the enzyme that generates H3meK79. Because γ-H2A was abundant in *SIC1*∆*P^HA^* cells that underwent DNA rereplication, and H3meK79 was likely to be abundant as well (van Leeuwen *et al.* 2002), these two histone modifications are necessary for the full checkpoint response, but not sufficient. In the mitotic cell cycle, particularly with respect to the G1 DNA damage checkpoint response, these modifications (as well as Rad6-Bre1 mediated histone H2B ubiquitylation required for H3meK79 generation) are important for Rad9 recruitment and Rad53 activation (Giannattasio *et al.* 2005; Javaheri *et al.* 2006; Hammet *et al.* 2007). In the checkpoint response that prevents DNA rereplication, neither *RAD9* nor *RAD53* appeared to be involved. While it is theoretically possible that Rad53 can be activated by a mechanism that cannot be detected by conventional means (electrophoretic mobility shift or *in situ* autophosphorylation), this possibility seems remote. In fact, our results are consistent with the fact that the Rad9-Rad53 axis is not involved in the meiotic recombination checkpoint that serves to prevent pachytene exit and progression through the meiotic divisions (Bishop *et al.* 1992; Lydall *et al.* 1996; Bailis and Roeder 2000). Of particular interest, however, is that residues in Dbf4, and presumably Sld3 as well, that are phosphorylated through Rad53 catalysis in the mitotic cell cycle to prevent late origin firing (Lopez-Mosqueda *et al.* 2010; Zegerman and Diffley 2010), also function in the meiotic response to prevent DNA rereplication. By contrast to the mitotic cell cycle observation (Zegerman and Diffley 2010), we found that the *mcm5-bob1* allele, which eliminates the requirement for the Dbf4-Cdc7 protein kinase in DNA replication initiation (Hardy *et al.* 1997), did not substitute for the *dbf4-4A* mutant allele as it did in the mitotic cell cycle studies. This distinction could reflect intrinsic differences in DNA replication regulation during the mitotic cell cycle and meiosis; alternatively, it could be due to the lowered CDK activity in our engineered cells.

**Figure 8:**
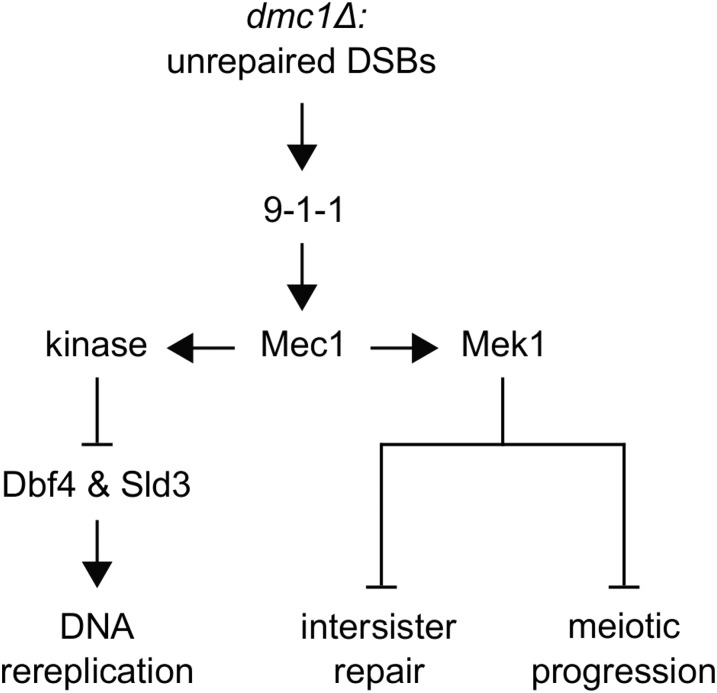
A meiotic recombination checkpoint response can inhibit DNA rereplication. This diagram based on our genetic analysis depicts certain key protein components in a pathway that leads from accumulation of unrepaired DSBs to inhibition of DNA rereplication in the *SIC1*∆*P^HA^* system. Also shown is an outline of the pathways that prevent intersister repair and meiotic progression in the normal checkpoint. See text for details.

Taken together, our results suggest a response in which a protein kinase that recognizes motifs in a similar manner to Rad53 is activated through a mechanism equivalent in many ways to the one that operates in response to DNA damage during the mitotic cell cycle (see Figure 8). One likely candidate for this kinase activity would be Mek1, given its structural similarity to Rad53 and its meiosis-specific expression (Rockmill and Roeder 1991; Leem and Ogawa 1992; Bailis and Roeder 2000). However, our data argue against this possibility because *SIC1*∆*P^HA^dmc1*Δ cells devoid of both *MEK1* and *RAD54*, designed to examine *MEK1* checkpoint function specifically, did not display DNA rereplication. Furthermore, while a peptide-based investigation into the phosphorylation site specificity of yeast protein kinases has placed Rad53 and Mek1 into the same largest group of five clusterings, the two kinases are not closely related in this regard, and exhibit a considerable difference in their degree of specificity (Mok *et al.* 2010). Another kinase with some physical similarity to Rad53 is Dun1, which, in response to genotoxic stress, operates downstream of Rad53 to regulate nucleotide pool levels and transcription (Allen *et al.* 1994; Zhao and Rothstein 2002). However, biochemical studies suggest that the two enzymes have different specificities (Zheng *et al.* 1993; Sanchez *et al.* 1997; Sidorova and Breeden 2003; Uchiki *et al.* 2004; Chen *et al.* 2007; Mok *et al.* 2010), and a comprehensive analysis of transcriptional regulation upon DNA damage indicates different targeting by Rad53 and Dun1 (Jaehnig *et al.* 2013). Therefore, evidence does not exist to indicate that Mek1 or Dun1 would likely catalyze phosphorylation of the same sites in Dbf4 and Sld3 as Rad53 does, suggesting that a different Rad53-like enzyme is present in meiosis that can influence initiation of DNA replication.

## Supplementary Material

Supplemental Material
