# Supplementary material for: Prevention of DNA Rereplication Through a Meiotic Recombination Checkpoint Response
Source: G3 (Bethesda). 2016 Sep 27;6(12):3869–81. doi: 10.1534/g3.116.033910 (PMC5144958; doi:10.1534/g3.116.033910)
Supplement: Supplemental Material [file supp_6_12_3869__index.html]

Prevention of DNA Rereplication Through a Meiotic Recombination Checkpoint Response — Supplemental Material 

# Prevention of DNA Rereplication Through a Meiotic Recombination Checkpoint Response

## Supplemental Material for Najor, *et al*, 2016

**Files in this Data Supplement:**

- Figure S1 - Quantification of DNA rereplication. (.eps, 703 KB)
- Figure S2 - *SIC1ΔPHA*-mediated DNA rereplication occurs in *rad54Δ* or *h2a-S129A* (γ-H2A-negative) cells. (.eps, 714 KB)
- Figure S3 - *PCH2* promotes *dmc1Δ*-dependent inhibition of *SIC1ΔPHA*-induced DNA rereplication. (.eps, 432 KB)
- Figure S4 - MYC-tagged Sic1 disappears during S phase in mitotic cells. (.eps, 190 KB)
- Figure S5 - *SIC1ΔPHA dmc1Δ h2a-S129A* cells are devoid of γ-H2A. (.eps, 956 KB)
- Figure S6 - Deletion of *RAD53*, but not *RAD9*, abolishes *SIC1ΔPHA*-induced DNA rereplication. (.eps, 352 KB)
- Figure S7 - Mutations in *DBF4* and *SLD3* or *MCM5* and *SLD3* do not prevent *SIC1ΔPHA*-induced DNA rereplication. (.eps, 476 KB)
